# The complete chloroplast genome of *Ranunculus muricatus* L. (Ranunculaceae): insights into genome features and evolutionary relationships

**DOI:** 10.1080/23802359.2025.2567460

**Published:** 2025-09-29

**Authors:** Chunfeng Wan, Junliu Song, Qingxia Chen, Han Liu, Hongqin Shang

**Affiliations:** ^a^ College of Agriculture and Bioengineering (Peony College), Heze University, Heze, Shandong Province, P. R. China; ^b^ The First Clinical School of Medicine, Zhengzhou University, Zhengzhou, Henan Province, P. R. China

**Keywords:** Comparative genomics, medicinal plant, phylogenetic evolution, polyanthemos

## Abstract

*Ranunculus muricatus* L. 1753, commonly known as spiny fruit buttercup, is a widely distributed medicinal plant. This study reported the first complete chloroplast genome of this species. The genome reveals a typical quadripartite structure, spanning 155,129 bp (LSC: 84,636 bp; SSC: 18,909 bp; IRs: 25,792 bp each) with 37.9% GC content. It was annotated 126 functional genes, including 81 protein-coding genes, 37 tRNA genes, and eight rRNA genes. Phylogenetic analysis showed that *R. muricatus* clustered a monophyletic group with *R. repens* within the section *Polyanthemos*. These findings support future research on its species identification, genetic diversity and phylogenetic evolution.

## Introduction

*Ranunculus muricatus* L. 1753, commonly referred to as spiny fruit buttercup, represents a fascinating subject of botanical and pharmacological research due to its widespread distribution across Asia, Australia, South America, and Europe (Hoa et al. 2021). This species belongs to the Ranunculaceae family, whose traditional medicinal applications have been documented across various cultures. It has been used to treat conditions ranging from respiratory ailments like cough and asthma to dermatological issues, including eczema and ringworm infections (Goo 2022).

Recent studies have lent credence to the traditional applications of *R. muricatus*, revealing significant antioxidant activity with compounds like muricazine (Aslam et al. 2013). The plant also exhibits enzyme inhibitory effects against acetylcholinesterase and α-glucosidase, along with anti-inflammatory and cytotoxic properties against various cancer cell lines (Khan et al. 2016; Raziq et al. 2017). These findings support its potential therapeutic applications in neurological, metabolic, and oncological disorders.

Despite extensive phytochemical research, genomic studies on *R. muricatus* remain limited. Investigating its chloroplast genome could provide valuable insights into evolutionary relationships, genetic adaptations, and so on. In this study, the chloroplast genome of *R. muricatus* was sequenced, assembled, and annotated. The structural characteristics of the chloroplast genome were analyzed in detail. Such genomic characterization would facilitate species identification, genetic diversity and phylogenetic evolution.

## Materials and methods

**Figure 1.**
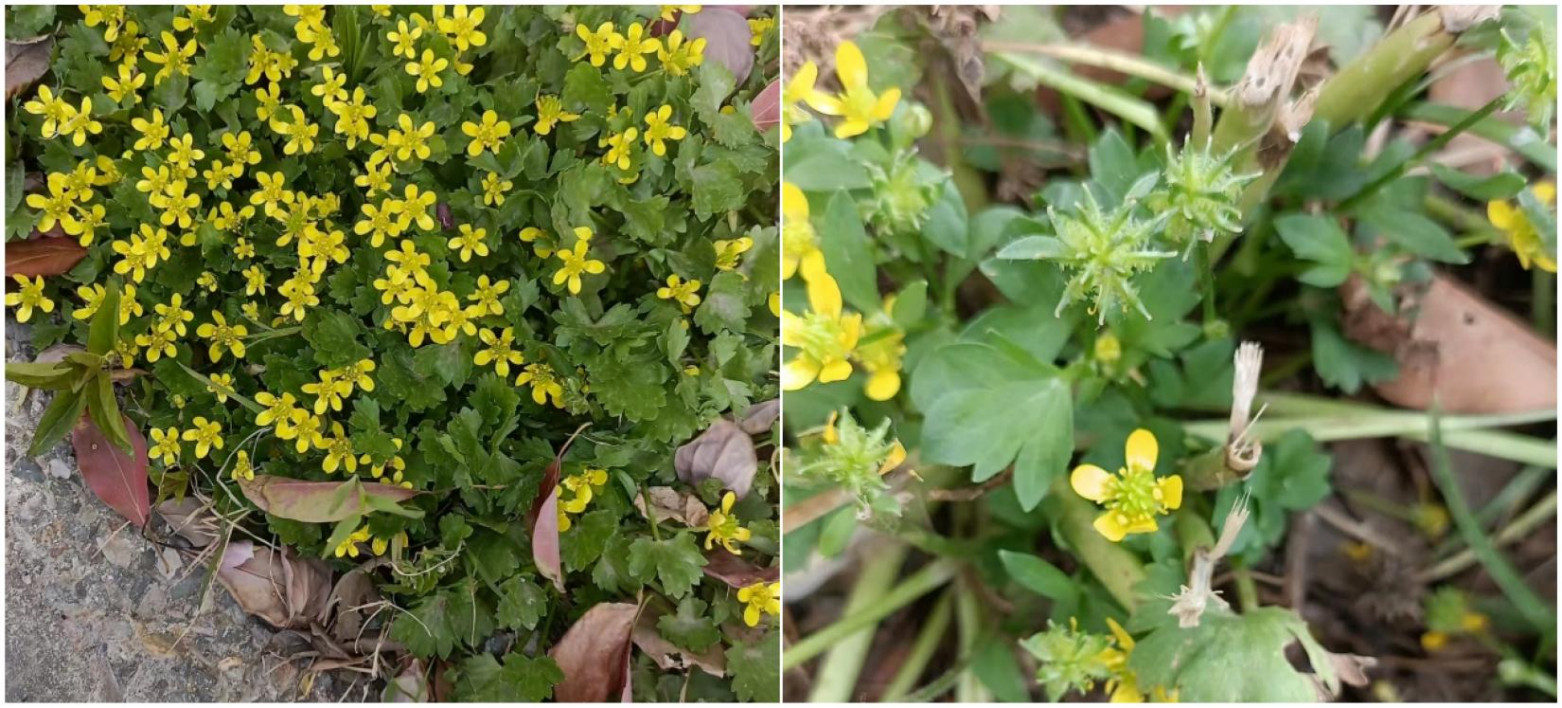
Field photographs of *ranunculus muricatus* showing habit and diagnostic features (photographer: Chunfeng Wan). An annual herb growing in roadsides and fields. Leaves simple, 3-lobed with serrated margins. Achenes flattened, with broad margins and 2 mm lateral spines. Flowering and fruiting: March-May.

Healthy fresh leaves of *R. muricatus* used for sequencing were obtained from Peony District, Heze City, Shandong Province, China (115.456555°E, 35.271757°N) (Figure 1). The voucher specimen was archived in the Herbarium of Heze University (contact: Chunfeng Wan, wancfeng@126.com) with an accession number of HZ20250103.

Total genomic DNA was isolated with the CTAB method (Doyle and Doyle 1987). Sequencing libraries were prepared using the Hieff NGS^®^ OnePot Pro DNA Library Prep Kit V4 (YEASEN, Shanghai, China), with genomic DNA enzymatically fragmented to an average size of 200–400 bp. High-throughput sequencing was subsequently carried out on the DNBSEQ platform with 150 bp paired-end read length. Fastp (v0.21.0) (Chen et al. 2018) was used to filter raw reads by removing those with >5% N bases, ≥50% low-quality bases (*Q* ≤ 5), adapter contamination, or PCR duplicates.

The complete chloroplast genome was assembled by GetOrganelle (v1.7.1) (Jin et al. 2020) with parameters “-R 50 -k 21,45,65,85,105 -P 1000000 -F embplant_pt”. The assembled genome was annotated by the online tool CPGAVAS2 (Shi et al. 2019) with Reference Dataset “43-plastomes” and manually adjusted through Apollo (v1.11.6) (Pontius 2018). Finally, the annotated chloroplast genome was submitted to GenBank with the accession number of PV744414. The online tool CPGview was utilized to exhibit the circular genome map of the novel chloroplast genome (Liu et al. 2023). The sequencing depth and coverage map was calculated by DrawSeqDepth (https://github.com/wlqg1983/DrawSeqDepth) with default parameters.

To analysis the phylogenetic position of *Ranunculus muricatus*, we retrieved chloroplast genomes from subgenera *Ranunculus* and *Auricomus* (taxonomic framework established by Hörandl and Emadzade (2012)). Our dataset included 19 *Ranunculus* species (excluding *R. muricatus* in this study) and two outgroups (*S. hexandrum* and *B. oiwakensis*). From the 22 chloroplast genomes, we extracted 64 shared protein-coding genes. The sequences were aligned using MAFFT (v7.505) (Katoh and Standley 2016), and a maximum-likelihood (ML) phylogenetic tree was reconstructed using IQ-TREE (v2.2.2.7) (Nguyen et al. 2015) employing the GTR+F + R2 model with 1,000 bootstrap replicates for nodal support.

## Results

**Figure 2.**
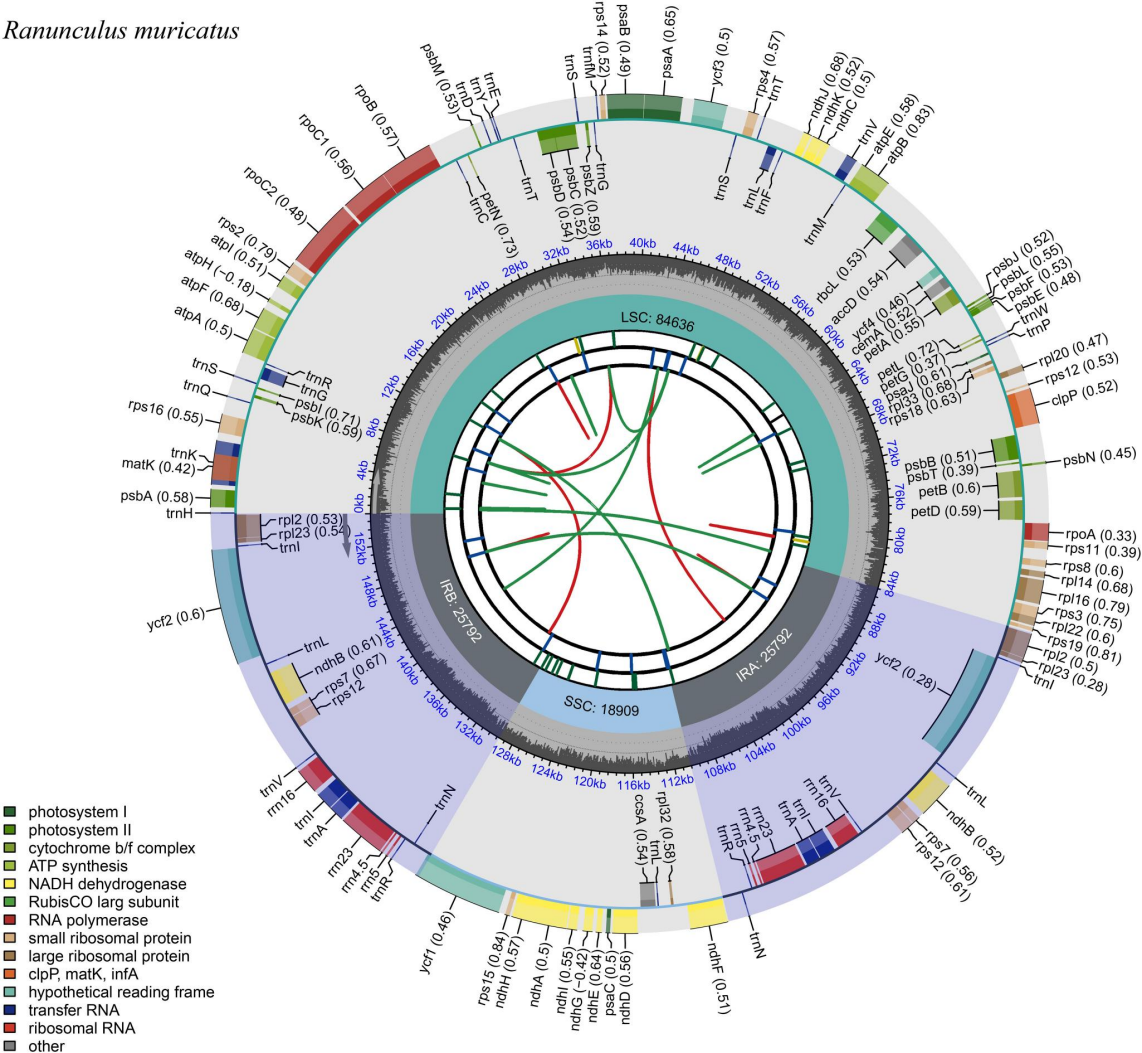
The chloroplast genome map of *Ranunculus muricatus.*

Following quality filtering and preprocessing, we obtained at least 22 Gb of whole-genome sequencing data (fastq format). These clean reads were used to assemble high-quality chloroplast genomes. The *R. muricatus* chloroplast genome sequence measured 155,129 bp long and presented a typical quadripartite structure (Figure 2). The assembled complete chloroplast genome exhibited an average sequencing depth of 1698.32×, with a minimum depth of 182× and a maximum depth of 2298× (Figure S1). The genome consists of two IR regions, each 25,792 bp in length, separated by a large LSC region of 84,636 bp and a small SSC region of 18,909 bp (Figure 2).

The chloroplast genome displayed the typical quadripartite structure consisting of a large single-copy (LSC) region, a small single-copy (SSC) region, and a pair of inverted repeats (IRa and IRb). Genes were color-coded by functional category and were distributed on both strands of the genome. The inner gray ring indicated the GC content across the genome. Functional categories included genes involved in photosystem I and II, cytochrome b6/f complex, ATP synthase, NADH dehydrogenase, RNA polymerase, ribosomal proteins (small and large subunits), Rubisco, tRNAs, rRNAs, and hypothetical chloroplast reading frames (*ycf*).

The chloroplast genome of *R. muricatus* annotation identified 126 genes (109 unique genes) (Table S1), comprising 81 protein-coding genes (75 are unique), 8 rRNA genes (4 are unique), and 37 tRNA genes (30 are unique). Additionally, the genome contained one trans-splicing gene (*rps*12) (Figure S2) and 22 (18 are unique) cis-splicing genes (14 protein-coding genes and 8 tRNA genes) (Figure S3). Trans-splicing gene *rps*12 had two 3′ tails each with one intron in the two IR regions. Except for the *rps*12, 19 genes had one intron and three genes have two introns. The chloroplast genome exhibited a varying GC content distribution, with an overall GC content of 37.9%. The highest GC content was found in the IR regions (43.5%), whereas the corresponding values were 36.0% and 31.0% for the LSC and SSC regions, respectively.

**Figure 3.**
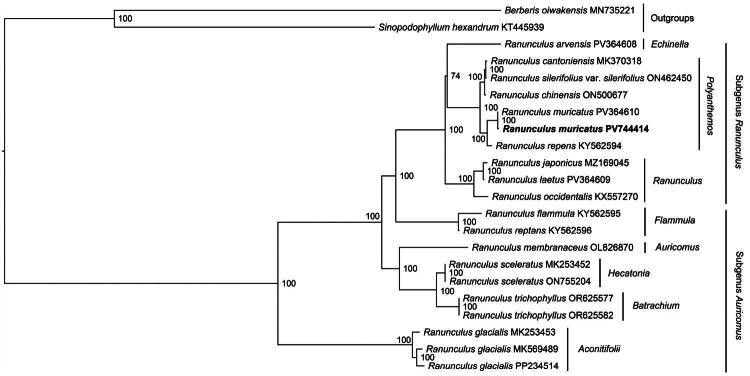
ML phylogeny of *ranunculus muricatus* and its close relatives. The bootstrap values based on 1000 replicates were shown on each node in the phylogenetic tree. The 22 species were downloaded from GenBank: *R. arvensis* (PV364608), *R. cantoniensis* (MK370318) (Li et al. 2019), *R. silerifolius* var. *silerifolius* (ON462450), *R. sardous* (OZ222637), *R. chinensis* (ON500677) (Ji et al. 2023), *R. muricatus* (PV744414, reported in this study and labeled by bold font), *R. muricatus* (PV364610), *R. repens* (KY562594), *R. japonicus* (MZ169045) (Zeng et al. 2021), *R. laetus* (PV364609), *R. occidentalis* (KX557270) (Ji et al. 2023), *R. flammula* (KY562595), *R. reptans* (KY562596) (Ji et al. 2023), *R. membranaceus* (OL826870) (Ji et al. 2023), *R. sceleratus* (ON755204) (Kim et al. 2023), *R. sceleratus* (MK253452) (Ji et al. 2023), *R. trichophyllus* (OR625582 and OR625577) (Ji et al. 2023), *R. glacialis* (MK253453, MK569489, and PP234514), *sinopodophyllum hexandrum* (outgroup, KT445939) (Meng et al. 2017), and *berberis oiwakensis* (outgroup, MN735221) (Xiao et al. 2020).

Phylogenetic analysis revealed a clear division of *Ranunculus* species into two major clades corresponding to subgenera *Ranunculus* and *Auricomus*, consistent with the taxonomic framework established by Hörandl and Emadzade (2012). Within subgenus *Ranunculus*, a well-supported monophyletic group was identified as section *Polyanthemos*, comprising *R. cantoniensis*, *R. silerifolius* var. *silerifolius*, *R. chinensis*, *R. muricatus*, and *R. repens*. Notably, the two *R. muricatus* accessions formed a distinct cluster sister to *R. repens* (Figure 3).

## Discussion and conclusion

We reported the primary characterization of the chloroplast genome of *R. muricatus* for the first time, which exhibited a typical annular tetrad structure with a size of 155,129 bp and 126 predicted genes. Our phylogenetic analysis revealed that *R. muricatus* clustered a monophyletic group with *R. repens. Ranunculus muricatus* exhibits the typical quadripartite chloroplast genome structure, with genome sizes ranging from 150,820 bp (*R. testiculatus*) to 158,314 bp (*R. trichophyllus*) (Ji et al. 2023). *Ranunculus muricatus* possessed the smallest chloroplast gene complement among *Ranunculus* species, with only 126 genes compared to the typical range of 126 (*R. sceleratus*; Kim et al. 2023) to 131 (*R. kadzusensis*; Choi et al. 2025).

*Ranunculus* is a taxonomically challenging genus due to the presence of morphologically similar species, making species identification difficult. Some researchers have suggested that *R. muricatus* should be included in the section *Polyanthemos* (Hörandl and Emadzade 2012). Phylogenetic analyses have proposed the inclusion of *R. muricatus* within section *Polyanthemos*, as suggested by Hörandl and Emadzade (2012). Our phylogenetic reconstruction strongly supported this classification, with *R. muricatus* forming a well-defined monophyletic clade within section *Polyanthemos*.

Current phylogenetic understanding of *Ranunculus* remains limited due to scarce chloroplast genome data. As noted by Qiao et al. (2023), robust phylogenetic reconstruction requires integrating nuclear and organelle genomes across more species. Future studies should expand sampling, generate high-quality genome sequences, and employ comprehensive phylogenetic analyses to resolve the complex evolutionary relationships of the *Ranunculus*.

## Supplementary Material

Supplemental Material

## Data Availability

The complete chloroplast genome sequence of *Ranunculus muricatus* in this study has been submitted to the NCBI database (https://www.ncbi.nlm.nih.gov) under the accession number PV744414. The associated BioProject, BioSample, and SRA numbers are PRJNA1146888, SAMN48789701, and SRR33755263.
