# Supplementary material for: The complete chloroplast genome of Ranunculus muricatus L. (Ranunculaceae): insights into genome features and evolutionary relationships
Source: Mitochondrial DNA B Resour. 2025 Sep 29;10(10):991–5. doi: 10.1080/23802359.2025.2567460 (PMC12481527; doi:10.1080/23802359.2025.2567460)
Supplement: Supplemental Material [file TMDN_A_2567460_SM0690.docx]

**Table** **S1.** **Genes** **annotated** **in** **the** ***Ranunculus*** ***muricatus*** **chloroplast** **genome**

| **Gene** **function** | **Gene** **type** | **Gene** **name** |
| --- | --- | --- |
| rRNA | rRNA genes | *rrn*4.5S(×2), rrn5S(×2), *rrn*16S(×2), *rrn*23S(×2) |
| tRNA | tRNA genes | *trn*H-GUG, *trn*K-UUU*, *trn*Q-UUG, *trn*S-GCU, *trn*G-UCC*, *trn*R-UCU, *trn*C-GCA, *trn*D-GUC, *trn*Y-GUA, *trn*E-UUC, *trn*T-GGU, *trn*S-UGA, *trn*G-GCC, *trn*fM-CAU, *trn*S-GGA, *trn*T-UGU, *trn*L-UAA*, *trn*F-GAA, *trn*V-UAC*, *trn*M-CAU, *trn*W-CCA, *trn*P-UGG, *trn*I-CAU(×2), *trn*L-CAA(×2), *trn*V-GAC(×2), *trn*I-GAU(×2)*, *trn*A-UGC(×2)*, *trn*R-ACG(×2), *trn*N-GUU(×2), *trn*L-UAG |
| Selfduplicate | Small subunit of ribosome | *rps*2, *rps*3, *rps*4, *rps*7(×2), *rps*8, *rps*11, *rps*12(×2)*, *rps*14, *rps*15, *rps*16*, *rps*18, *rps*19 |
|  | Large subunit of ribosome | *rpl*2(×2)*, *rpl*14, *rpl*16*, *rpl*20, *rpl*22, *rpl*23(×2), *rpl*32, *rpl*33 |
|  | DNA dependent RNA polymerase | *rpo*A, *rpo*B, *rpo*C1*, *rpo*C2 |
| Photosynthesis | Subunits of NADH-dehydrogenase | *ndh*A*, *ndh*B(×2)*, *ndh*C, *ndh*D, *ndh*E, *ndh*F, *ndh*G, *ndh*H, *ndh*I, *ndh*J, *ndh*K |
|  | Subunits of photosystem Ⅰ | *psa*A, *psa*B, *psa*C, *psa*J |
|  | Subunits of photosystem Ⅱ | *psb*A, *psb*B, *psb*C, *psb*D, *psb*E, *psb*F, *psb*I, *psb*J, *psb*K, *psb*L, *psb*M, *psb*N, *psb*T, *psb*Z, *ycf*3** |
|  | Subunits of cytochrome b/f complex | *pet*A, *pet*B*, *pet*D*, *pet*G, *pet*L, *pet*N |
|  | Subunits of ATP synthase | *atp*A, *atp*B, *atp*E, *atp*F*, *atp*H, *atp*I |
|  | Large subunit of rubisco | *rbc*L |
| Other genes | Maturase | *mat*K |
|  | Protease | *clp*P** |
|  | Envelope membrane protein | *cem*A |
|  | Subunit of Acetyl-CoA-carboxylase | *acc*D |
|  | c-type cytochrom synthesis gene | *ccs*A |
| Genes of unknown functions | Open Reading Frame | *ycf*1, *ycf*2(×2), *ycf*4 |
| Note: Numbers in the parentheses represent the number of copies. *, **: indicte one and two introns, respectively. | | |


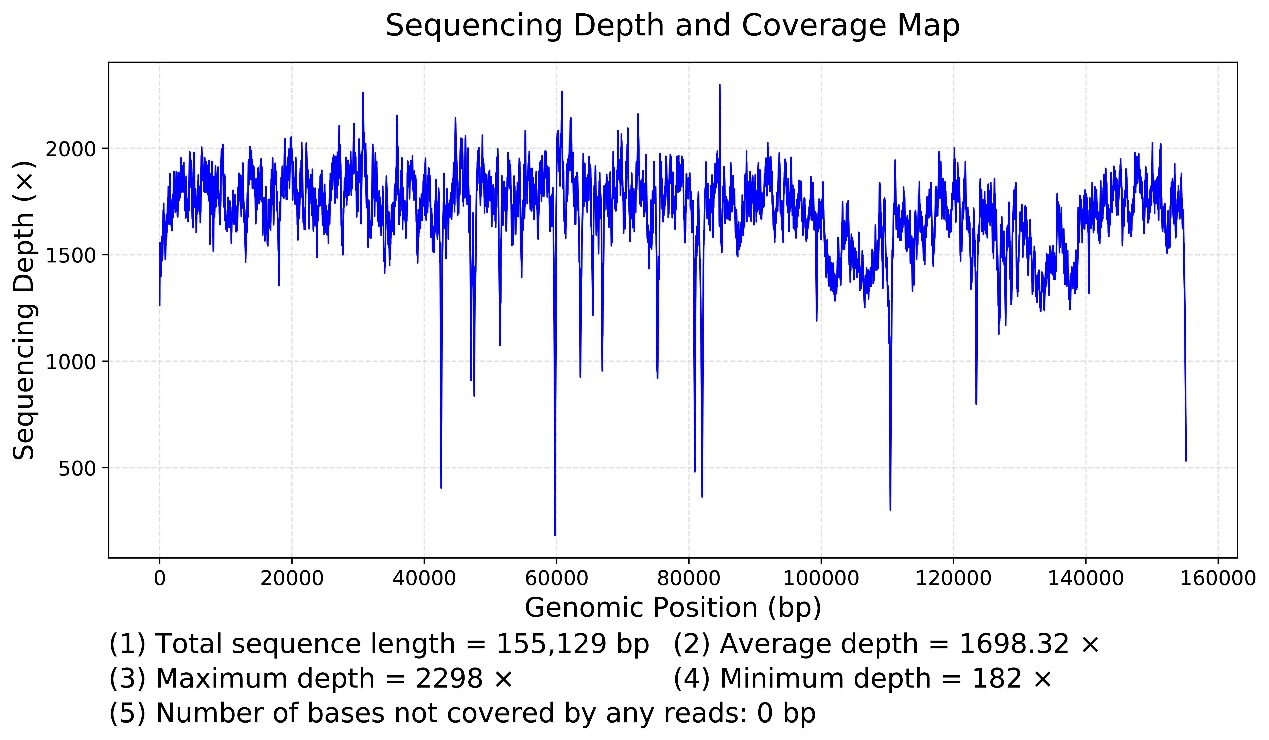


**Figure** **S1.** **The** **coverage** **depth** **of** **the** **chloroplast** **genome** **assembly** ***Ranunculus*** ***muricatus*.**


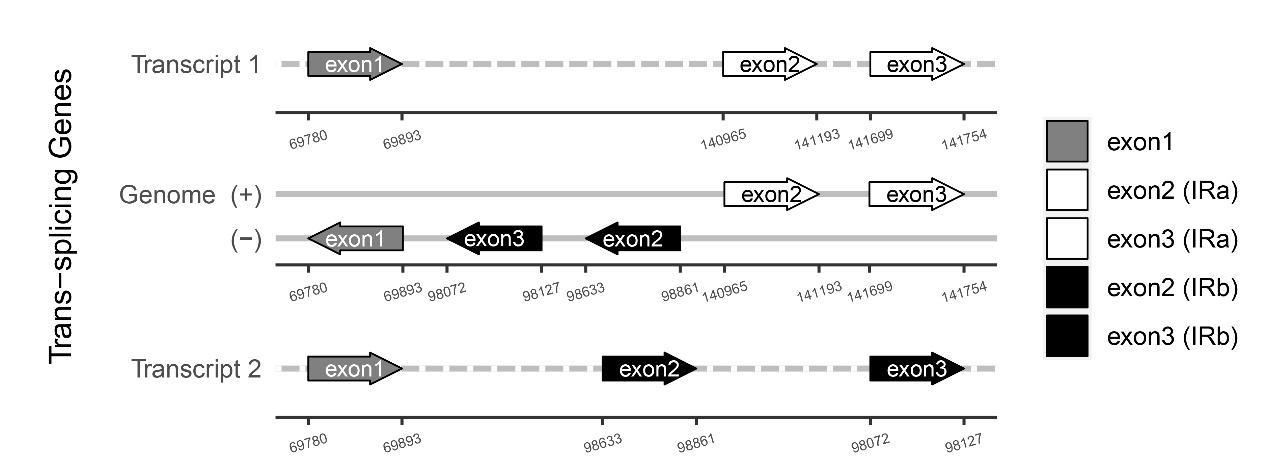


**Figure** **S2.** **The** **map** **of** **Trans-splicing** **genes** **(*rps*12)** **of** ***Ranunculus*** ***muricatus*.** The exons are shown in black and grey, and the introns are shown in white. The arrow indicates the sense direction of the gene.


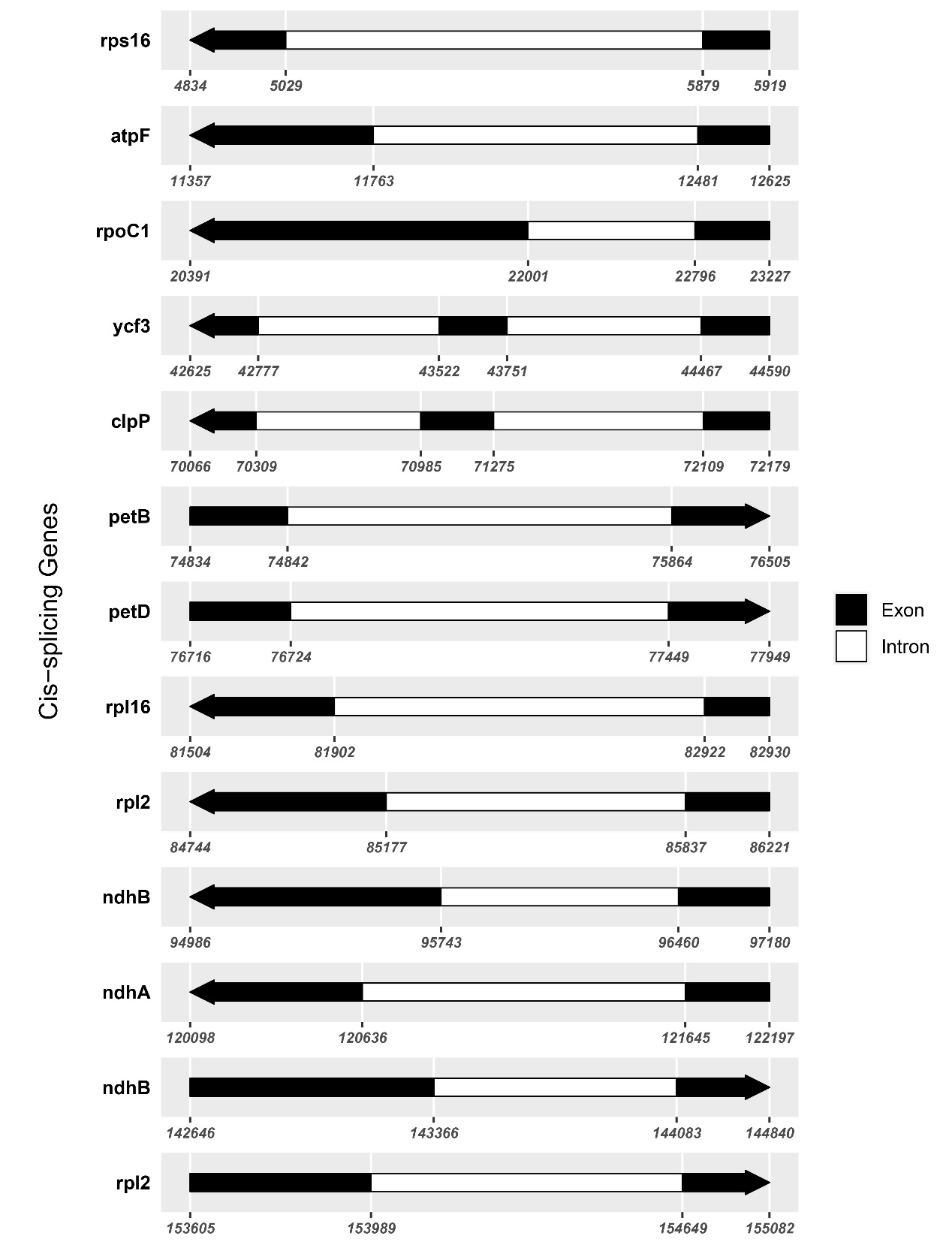


**Figure** **S3.** **Schematic** **map** **of** **the** **cis-splicing** **genes** **in** **the** **chloroplast** **genome** **of** ***Ranunculus*** ***muricatus*.** The gene names are shown on the left, and the gene structures are on the right. The exons are shown in black and the introns are shown in white. The arrow indicates the sense direction of the gene. The lengths of exons and introns are not drawn to scale.
